# First Reported Case of Intracardiac Kimura Disease

**DOI:** 10.1016/j.jaccas.2025.105215

**Published:** 2025-09-05

**Authors:** Andrew D. Wisneski, Jamal S. Rana, Eugene Fan, Jessica A. Tashjian, Patty Pei-Chang Chen, John L. Chen, Paul M. LaPunzina, Ahmad Y. Sheikh

**Affiliations:** a Division of Cardiothoracic Surgery, Department of Surgery, University of California San Francisco, San Francisco, California, USA; b Department of Cardiology, Kaiser Oakland Medical Center, Oakland, California, USA; c Division of Cardiology, University of Illinois College of Medicine, Chicago, Illinois, USA; d Department of Cardiovascular Anesthesia, Kaiser San Francisco Medical Center, San Francisco, California, USA; e Department of Rheumatology, Kaiser Oakland Medical Center, Oakland, California, USA; f Department of Cardiac Surgery, Kaiser San Francisco Medical Center, San Francisco, California, USA

**Keywords:** cardiac mass, Kimura disease, pulmonary valve replacement, right ventricular outflow tract

## Abstract

**Background:**

Kimura disease is a rare inflammatory condition that typically manifests with subcutaneous nodules of the head and neck. This is the first documented case of intracardiac Kimura disease.

**Case Summary:**

A 57-year-old woman presented with a heart murmur and dyspnea. Echocardiography uncovered a 3-cm mass obstructing the right ventricular outflow tract. The mass was resected, and the pulmonary valve was replaced. Pathology yielded the diagnosis of Kimura disease.

**Discussion:**

Treatment of Kimura disease may include surgery, steroids, and radiation therapy. There is potential for disease recurrence.

**Take-Home Messages:**

This is the first reported case of intracardiac Kimura disease, an inflammatory condition usually involving the head and neck. The working diagnosis for the right ventricular outflow tract mass was myxoma until final pathologic analysis was obtained. Our patient underwent surgical resection without steroids or radiation. Active surveillance with patient follow-up and surveillance echocardiography over 1 year demonstrated no recurrence.

## Visual Summary

**Figure:**
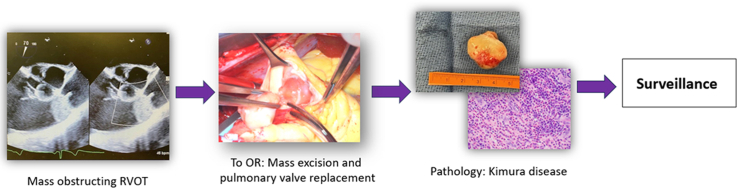

**Visual Summary:**
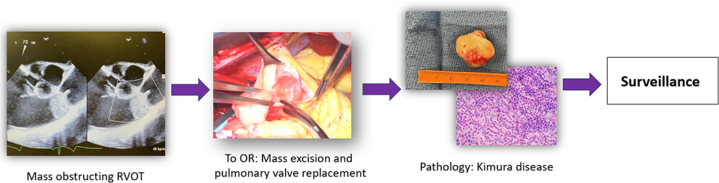
Imaging, Excision, and Pathology OR = operating room; RVOT = right ventricular outflow tract.

## History of Presentation

A 57-year-old female ballet instructor presented with worsening dyspnea on exertion in the setting of known, mild pulmonary stenosis of unknown etiology. Given her worsening symptoms, an extensive work-up was done, and she was found to have a 3-cm obstructing mass in the right ventricular outflow tract (RVOT) with resultant moderate pulmonary stenosis. On examination, she appeared well with no peripheral edema or skin changes. Vital signs were within normal limits. An infectious work-up was negative, and there were no other indications of systemic illness or malignancy.

**Take-Home Messages**

• This is the first reported case of intracardiac Kimura disease, an inflammatory condition usually involving the head and neck. The working diagnosis for the RVOT mass was myxoma until final pathologic analysis was obtained.
• Our patient underwent surgical resection without steroids or radiation. Active surveillance with patient follow-up and surveillance echocardiography over 1 year demonstrated no recurrence.

## Past Medical History

The patient's medical history included Graves disease, which was treated with iodine ablation a decade earlier. She was maintained on thyroid replacement therapy at the time of presentation.

## Differential Diagnosis

The differential diagnosis for cardiac mass in the RVOT includes benign tumors such as myxomas and rhabdomyomas, with myxoma being the most prevalent primary cardiac mass. Malignant tumors include sarcomas and lymphomas. Thrombi may be seen secondary to malignancy or a hypercoagulable state. Other possibilities include infectious endocarditis, tuberculomas, or rheumatologic etiologies such as Libman-Sacks endocarditis, Behçet disease, sarcoidosis, IgG_4_-related disease, and anti-neutrophil cytoplasmic antibody vasculitis.

## Investigations

Routine screening laboratory tests all were within normal limits, with serum creatinine 0.78 mg/dL, white blood cell count 4.4 cells/μL, hemoglobin 14.2 g/dL, and platelet count 273 × 10^9^/L. Cardiac troponin and B-type natriuretic peptide were normal, and the patient was euthyroid. Chest x-ray was within normal limits, COVID-19 test was negative, and electrocardiogram demonstrated normal sinus rhythm.

**Figure 1:**
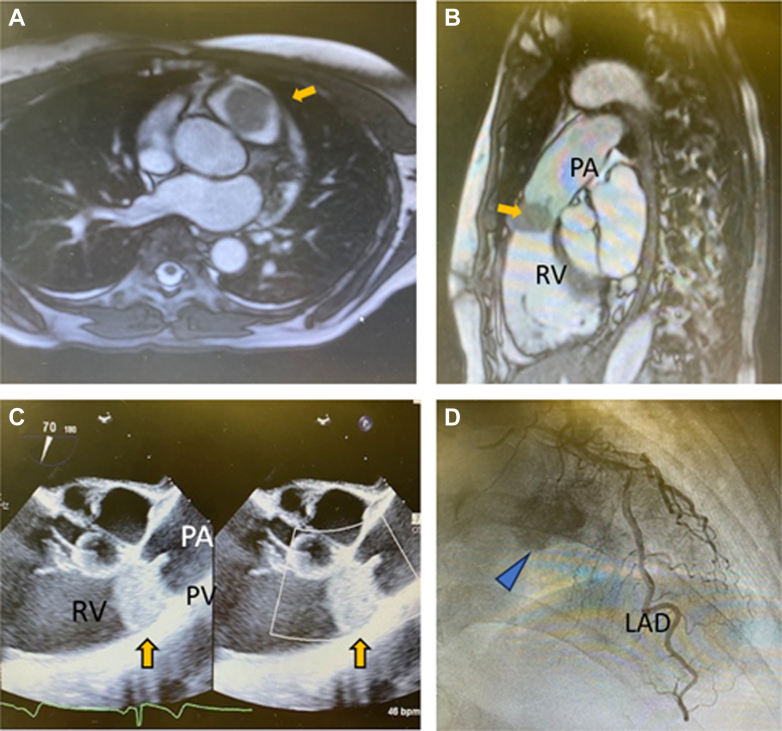
Preoperative Imaging (A) Cardiac magnetic resonance (axial slice) with mass (yellow arrow) in right ventricular outflow tract. (B) Sagittal image of cardiac magnetic resonance showing mass (yellow arrow) with close association of the pulmonary valve (PV). (C) Echocardiography demonstrating mass and involvement with PV with right ventricular outflow tract obstruction. (D) On left heart catheterization, the mass opacifies (blue arrow) with contrast agent injected into the left main coronary artery. LAD = left anterior descending coronary artery; PA = pulmonary artery; RV = right ventricle.

Echocardiography revealed a smooth, round 2 × 3 cm mobile mass obstructing the RVOT at the level of the pulmonary valve (Figure 1). Moderate pulmonary stenosis was present with a mean gradient of 21 mm Hg (Videos 1 and 2). Left ventricle ejection fraction was 65%. Cardiac magnetic resonance found the mass to exhibit heterogeneous signal enhancement and further confirmed that the mass appeared adherent to the pulmonary valve (Figure 1, Video 3). Coronary angiography yielded no significant coronary artery disease but showed that the mass derived blood supply from the left coronary artery (Figure 1, Videos 4 and 5). This key feature suggested a chronic process and helped exclude entities such as endocarditis or thrombus. Surgical resection was planned.

## Management

**Figure 2:**
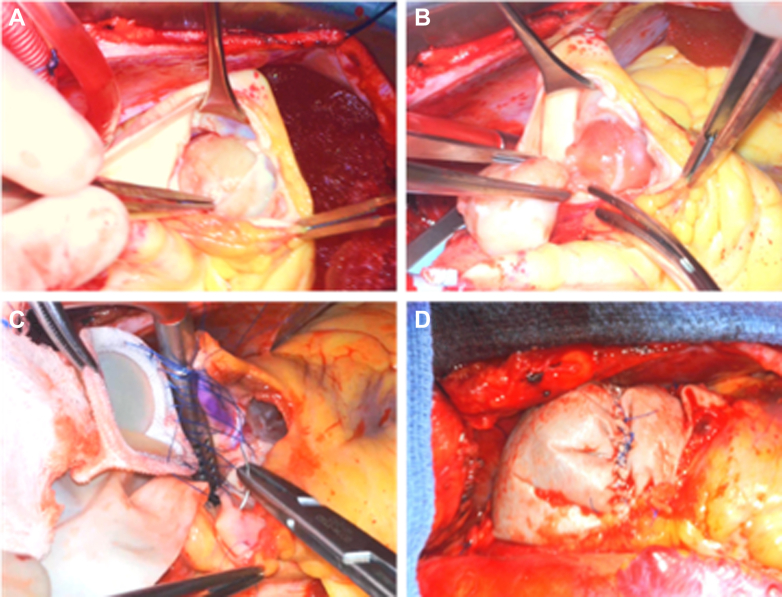
Operative Resection of Mass (A) Longitudinal incision over the main pulmonary artery coursing over the pulmonary valve annulus reveals the mass. (B) Mass is removed with the left pulmonary valve leaflet. (C) Pulmonary valve prosthesis sutured to the annulus. (D) Right ventricular outflow tract reconstructed with bovine pericardial patch.

After median sternotomy and initiation of cardiopulmonary bypass, the aorta was cross-clamped, and the heart was arrested with cardioplegia. A longitudinal incision was made on the main pulmonary artery traversing the pulmonary valve annulus onto the RVOT (Figure 2). The mass was found on the ventricular aspect of the pulmonary valve, consistent with preoperative imaging. Whereas the right and anterior leaflets of the pulmonary valve were free, the left leaflet was adherent to the mass and could not be separated. The left leaflet of the pulmonary valve was detached from the annulus, and several attachments from the RVOT to the mass were freed. The specimen was removed ensuring that several millimeters of tissue margin was obtained (Video 6).

The pulmonary valve was replaced with a 27-mm Inspiris bovine pericardial tissue valve (Edwards LifeSciences), with pericardial patch reconstruction of the pulmonary artery (Figure 2). Intraoperative echocardiography demonstrated a well-seated pulmonary valve prosthesis with normal gradients.

## Outcome and Follow-Up

**Figure 3:**
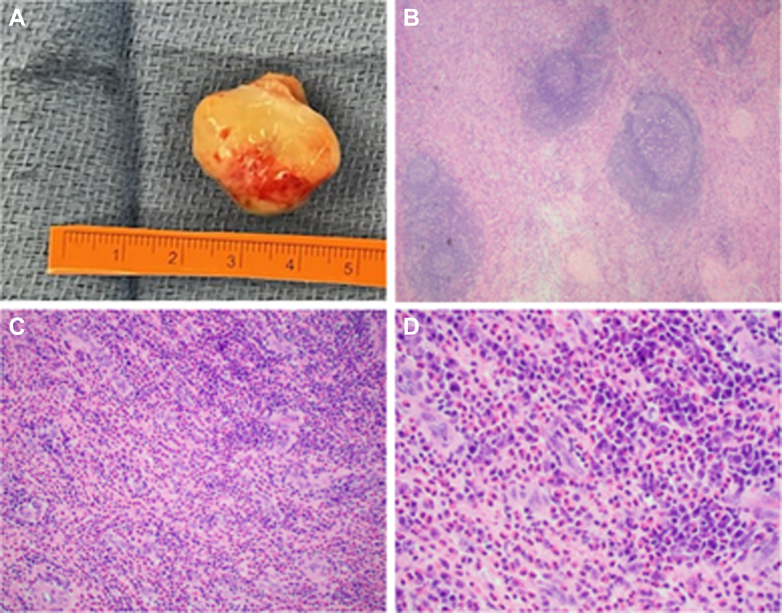
Specimen and Histology (A) Gross specimen from the operating room. (B) Pathology slide with histology demonstrating reactive follicles with expansion of interfollicular areas (magnification ×5). (C) Histology demonstrates mixed cellular infiltrate in interfollicular areas (magnification ×20). (D) Many eosinophils mixed with plasma cells (magnification ×40).

The patient's recovery was uneventful, and she was discharged home on postoperative day 5. Final pathologic analysis of the mass yielded a diagnosis of Kimura disease (Figure 3). Histology revealed the hallmark findings of reactive follicles, vascular proliferation with mixed cellular infiltrate in interfollicular areas, and eosinophilic infiltration.^1^^,^^2^

At 1-month follow-up, the patient was doing well with no shortness of breath. Echocardiography revealed a normally functioning pulmonary valve prosthesis. A multidisciplinary team (rheumatology, cardiology, and internal medicine) was assembled to manage the patient postoperatively. Routine surveillance was planned, and the decision was made not to pursue radiation therapy. At 12-month follow-up, the patient remained asymptomatic with serial echocardiograms demonstrating no evidence of recurrence.

## Discussion

Kimura disease is a rare inflammatory condition and is not typically considered as part of the differential diagnosis for a cardiac mass. First described in 1937 by Kim and Szeto^3^ in China as eosinophilic hyperplastic lymphogranuloma, it eventually received its eponymous designation when described by Kimura et al.^4^ Kimura disease is typically seen in people of Asian descent, presenting at 20 to 40 years of age with a predominantly male predisposition of 3 to 4:1 (male-to-female ratio). Patients usually present with subcutaneous nodules in the head and neck region with regional lymphadenopathy. Laboratory tests often demonstrate peripheral eosinophilia and elevated immunoglobulin E.^1^^,^^2^^,^^5^^,^^6^ The gold standard for diagnosis remains biopsy of the nodule or lymph node.

Two prior reports describe cardiac sequelae related to systemic disease involvement of Kimura disease: 1 case of nonischemic cardiomyopathy and 1 case of complete heart block with associated glomerulonephritis.^6^^,^^7^ Notably, both cases occurred in the setting of significant peripheral eosinophilia (>1.5 × 10^9^/L), which itself may be associated with end-organ dysfunction.^8^ To our knowledge, our report is the only documented case of primary manifestation of Kimura disease as an intracardiac mass; the patient lacked any of the typical subcutaneous findings, had no peripheral eosinophilia, and had normal renal function. With the rarity of Kimura disease and the literature comprising only a handful of case series and reports, the unique presentation of this case raises the possibility that primary manifestation as an inflammatory mass in an internal organ may be the initial and only presentation. Unless careful pathologic analysis is performed, it may be misattributed to another disease entity. Identification of additional intracardiac Kimura disease may help provide insight into this disease phenotype and pathophysiology.

Treatment for Kimura disease may entail surgical excision of nodules or affected structures, administration of steroids or immunosuppressants, and radiation therapy. Recurrence rates of Kimura disease range from 30% to 60%, underscoring the importance of surveillance.^1^ However, these data are from patients without cardiac involvement. Although Kimura disease appears to have a benign course, intracardiac recurrence could carry substantial risk given the potential need for redo surgery.

Steroid therapy was not given, as the patient did not have any extracardiac manifestations of the disease. Radiation therapy was not initiated given its potential to cause early deterioration of the bioprosthetic valve and lack of data for efficacy in this rare presentation.

In review of this case, additional factors bear discussion. Systemic imaging (magnetic resonance imaging or combined positron emission tomography/computed tomography) to search for radiographic evidence of extracardiac foci of Kimura disease was not done. The patient's history and physical examination, aside from the cardiac symptoms, did not raise suspicion for other disease locations. At the time of this writing, the patient has had 12 months of postoperative follow-up. Given Kimura disease recurrence rates of 30% to 60%, long-term follow-up will remain crucial to the successful management of this patient and early detection of any recurrence.

This is the only report of primary intracardiac Kimura disease to date. If additional cases can be identified, insight may be obtained into the pathophysiology of this disease phenotype.

## Conclusions

This is the first reported case of intracardiac Kimura disease. This is a rare inflammatory condition, and treatment may entail surgery, steroids, and/or radiation therapy. Given recurrence potential, careful follow-up is recommended. This case highlights how very rare entities can masquerade as more common ones and the need for detailed pathologic analysis for seemingly routine cardiac masses.

## Funding Support and Author Disclosures

The authors have reported that they have no relationships relevant to the contents of this paper to disclose.

## Appendix

Video 1 Coronary Angiography (View 1) Coronary angiography demonstrating blood supply to mass.

Video 2 Coronary Angiography (View 2) Coronary angiography demonstrating blood supply to mass.

Video 3 Cardiac Magnetic Resonance Cardiac magnetic resonance showing mass in right ventricular outflow tract.

Video 4 Echocardiography Transesophageal echocardiography demonstrating mass in right ventricular outflow tract.

Video 5 Echocardiography With Doppler Transesophageal echocardiography with color Doppler mode demonstrating pulmonary valve stenosis from mass in right ventricular outflow tract.

Video 6 Operative Resection of Mass Longitudinal incision on main pulmonary artery and excision of mass with pulmonary valve leaflet.
